# Control over Drug Acquisition, Preparation, and Injection: Implications for HIV and HCV Risk among Young Female Injection Drug Users

**DOI:** 10.1155/2013/289012

**Published:** 2013-06-03

**Authors:** Karla D. Wagner, Jennifer Jackson Bloom, Susan Dodi Hathazi, Bill Sanders, Stephen E. Lankenau

**Affiliations:** ^1^Division of Global Public Health, Department of Medicine, University of California, San Diego, 9500 Gilman Drive, MC 0849, La Jolla, CA 92093, USA; ^2^Division of Adolescent Medicine, Children's Hospital Los Angeles, Keck School of Medicine, University of Southern California, 4650 Sunset Boulevard, MS 2, Los Angeles, CA 90027, USA; ^3^Department of Nursing, Psychiatry, New York University Langone Medical Center, 550 First Avenue, New York, NY 10016, USA; ^4^School of Criminal Justice and Criminalistics, California State University, Los Angeles, 5151 State University Drive, Los Angeles, CA 90032, USA; ^5^Department of Community Health and Prevention, Drexel University School of Public Health, 1505 Race Street, Bellet Building, Philadelphia, PA 19102-1192, USA

## Abstract

Young female injection drug users (IDUs) are at risk for HIV/HCV, and initiating the use of a new drug may confer additional and unexpected risks. While gender differences in the social context of injection drug use have been identified, it is unknown whether those differences persist during the initiation of a new drug. This mixed-methods study examined the accounts of 30 young female IDUs in Los Angeles, CA, USA from 2004 to 2006, who described the social context of initiating injection drug use and initiating ketamine injection. The analysis aimed to understand how the social context of young women's injection events contributes to HIV/HCV risk. Women's initiation into ketamine injection occurred approximately 2 years after their first injection of any drug. Over that time, women experienced changes in some aspects of the social context of drug injection, including the size and composition of the using group. A significant proportion of women described injection events characterized by a lack of control over the acquisition, preparation, and injection of drugs, as well as reliance on friends and sexual partners. Findings suggest that lack of control over drug acquisition, preparation, and injection may elevate women's risk; these phenomena should be considered as a behavioral risk factor when designing interventions.

## 1. Introduction

Though considerable declines in new HIV infections have been observed since the late 1980s, injection drug use continues to account for an estimated 12% of incident HIV infections in the United States [1]. Recent behavioral surveys suggest that approximately 30% of IDUs have shared syringes or other injection equipment in the last year [2]. The social context of injection drug use has been shown to differ for men and women, which has been posited to explain gender differences in risk for HIV/HCV infection among IDUs [3, 4]. These social differences include the role relation of the injecting partner (women more frequently use drugs with individuals with whom they have a relationship, usually a sex partner [5]); the drug use behavior of women's social network contacts (female IDUs' social networks tend to have more “hard drug” users (e.g., heroin and cocaine) and IDUs than male IDUs' networks [4]); and the greater degree to which women's drug, sex, and friend networks overlap [3, 6]. 

Women's risk is also elevated because their access to drugs, injection paraphernalia, and other resources are often controlled or determined by their sex partners and/or others within their social network [5, 7–9]. Finally, and perhaps most importantly in terms of injection-related risk, women often relinquish control over the actual preparation and injection processes to others, usually their male sex partners [10]. Not feeling in control over the injection event has been associated with unsafe injection practices among women [11], and HIV infection has been shown to be almost twice as high among drug users who require help injecting [12]. 

Initiation into injection drug use is a significant event with well-documented health risks [13–15]. However, there is far less information on the effects of experimentation with the injection of a new drug and how behaviors associated with the injection process change over time. There is some evidence that the context of initiation of a new drug (i.e., ketamine) among established IDUs can introduce new and unexpected risks for HIV/HCV. Ketamine is a legally manufactured, dissociative anesthetic that was originally developed for surgical use [16], but which has emerged as a recreational drug [17]. Young people who inject ketamine have been found to be a particularly high-risk group of IDUs who tend to engage in sequential and/or simultaneous polydrug use [18, 19] and a number of risky injection practices, including sharing injection paraphernalia [20]. Ketamine can usually be obtained in two forms on the street: powder or liquid. Powder ketamine is often used intranasally, but can also be mixed with water and injected. Liquid ketamine is generally sold in pharmaceutically sealed vials with lids that are designed to be pierced by hypodermic syringes and can be injected intramuscularly or intravenously.

While sometimes addressed as separate risk factors in various reports, this study examines five interrelated features of the social context of injection drug use that may have implications for increased risk of exposure to HIV and HCV among young female IDUs: characteristics of the using group, control over access to drugs, control over access to injection paraphernalia, control over preparation of drugs, and control over injection of drugs. In particular, we examine how the context of injection drug use changes among young female IDUs by comparing the first injection event of any drug (e.g., heroin, cocaine, and methamphetamine) with the first injection of ketamine, which occurred later in the injection career. Last, we examine implications for HIV/HCV risk and identify opportunity for risk reduction and targeted HIV and HCV prevention interventions among young female IDUs.

## 2. Design and Method

### 2.1. Sampling

The current study examines a sample of 30 young female IDUs recruited as part of a larger, three-site mixed-methods study designed to investigate the role of ketamine injection in shaping HIV risk among young IDUs. The larger study included 222 young ketamine IDUs recruited from three data collection sites: New York City, Los Angeles, and New Orleans. All data were collected between March 2004 and June 2006, and respondents completed a single cross-sectional interview in each site. Sampling and data collection procedures were the same in all three sites and were approved by local institutional review boards. Data for the current analysis were drawn from the Los Angeles site in order to reduce the variability introduced by differences in geographic locations. 

Data collection began with a community assessment process (CAP) [21], in which ethnographers conducted interviews with key informants and community members (e.g., directors of local service agencies for youth and IDUs) in order to identify locations for participant recruitment. Based on the information provided in the CAP, ethnographers recruited young ketamine injectors using a combination of chain referral sampling [22, 23] and targeted sampling [24]. Both sampling strategies are nonrandom methods that are effective in sampling hidden populations for whom no population estimates exist. In the Los Angeles area, ethnographers focused recruitment efforts in the cities of Hollywood, Santa Monica, and Venice Beach.

### 2.2. Enrollment

Eligible individuals were 16 to 29 years old and had injected ketamine at least once within the past two years. Ethnographers first administered a series of screening questions that asked about health behaviors, recent drug use, and history of homelessness in order to conceal the eligibility criteria. Eligible individuals provided informed consent and completed a single cross-sectional interview that lasted approximately one hour. Participants were compensated with $20 cash payments and referral information for local needle exchanges, health clinics, homeless shelters, and other service provider information. Because this analysis included data from both the first injection event and the first injection of ketamine, women who initiated injection drug use with ketamine were not included in this analysis. One additional woman was excluded due to inconsistent responses, yielding an analytical sample of 30 women.

### 2.3. Measures

The interview guide contained a mix of structured, closed-ended questions and open-ended, qualitative questions. Interviews were conducted using a computer-assisted interview, programmed in Questionnaire Development Software (QDS) [25] and were digitally recorded. Closed-ended questions included standard demographic items (e.g., age, race/ethnicity, self-reported sexual identity, education level, homelessness, employment history, history of drug treatment, and mental health care, and criminal justice involvement). HIV and HCV status were assessed using self-report of having been tested and results of the most recent test, if applicable (positive, negative, and unknown). Type of drug injected, number of people present at the injection event, and whether the injection event was planned (yes/no) were also collected with closed-ended questions. Syringe source was assessed using a single question that asked whether the participant obtained the syringe herself from a syringe exchange or pharmacy (primary source) or from someone else (secondary source). Closed-ended questions also assessed the participants' relationship with others involved in the preparation and injection of the drug. Open-ended questions asked participants to describe the context of injection events. For example, participants were asked about the geographic location, physical setting (i.e., in a building, outside, etc.), social setting (i.e., who else was present), why they injected, how they obtained the drugs, and a detailed description of the procedures for preparing and injecting the drug. Interviewers used follow-up probes to elicit more information about details of the injection events, as appropriate.

### 2.4. Analysis

Data for the current analysis were drawn from two interview modules, which asked participants about two injection events: the initiation of drug injection with any drug and the initiation of ketamine injection. To describe how women's injection behaviors changed over time, we compared circumstances at the first injection of any drug to those at the initiation of ketamine, which occurred an average of two years later.

Quantitative data were analyzed using SPSS to generate descriptive statistics. Digital recordings were transcribed in their entirety. The qualitative aspects of this analysis occurred in a three-step process. First, transcripts were read and a series of “memos” was developed, which documented initial impressions [26]. Second, based on the initial reading of the transcripts and some a priori understanding of themes that have been identified in the literature, a list of “open codes” was developed [27]. These codes were applied to segments of text using the ATLAS.ti program [28], which allows the analyst to code and organize text. When new codes emerged from the transcripts during the coding process, the emergent codes were added to the existing list, and all transcripts were reviewed again to ensure the coding of relevant passages. Third, reports were generated that contained blocks of coded text from all the interview transcripts, and the results were compared for the two injection events (first injection of any drug and first injection of ketamine) to identify similarities and differences. 

## 3. Results

### 3.1. Demographics

Participants had a median age of 21 years and were mostly white (Table 1). Sixty percent of women identified as heterosexual, while 40% identified as bisexual. All women reported being currently homeless. Women reported high rates of involvement with the criminal justice system, high rates of HIV and HCV testing, and low rates of employment in either full or part time work. No women reported being HIV positive, and 33% reported being HCV positive. In terms of drug use, about half of women reported that heroin was the first drug they ever injected, while just over one-quarter initiated with methamphetamine (Table 2).

### 3.2. Using Group

At the time of the first injection, women reported that they injected with a median of 2 other people (range 1–5). In their descriptions of the first injection event, most women (about two-thirds) noted the presence of friends, sometimes described as members of their “street family.” Almost half also reported the presence of a sexual partner (i.e., boyfriend, girlfriend, fiancé, or husband) at their first injection episode. Often, using groups were composed of mixed groups of friends and sex partners. Most women initiated injection of any drug with a using group that comprised older individuals, though some reported groups that were approximately of their same age. No women reported strangers as part of their using group, and only two reported injecting alone.

An average of two years elapsed (range 0–10 years) between the first injection of any drug and first injection of ketamine. While the median size of using groups remained the same, the range increased (median = 2, range 1–15). The composition of women's using groups at their first ketamine injection differed from what they described at their first injection event. Rather than mostly older groups, women described an almost equal number of older and same age individuals, with a large proportion of mixed age groups. While slightly fewer women reported having friends present, there were more relationship types reported, including “sugar daddy,” “random people at a party,” and “drug dealers.” Only about one-third reported that sex partners were present. Again, only two women reported injecting alone. 

### 3.3. Access to Drugs

At the time of their first injection, only one in five women paid for the drug that they injected (Table 2). Half said that their first injection was planned, or that they had deliberately sought out the opportunity. Several women reported that they “never” pay for their own drugs, and only a few women reported having a direct connection to the drugs (i.e., to a dealer); the majority of women acquired their drugs through someone else. Most of these intermediaries were men, either friends or boyfriends. Only one woman made explicit reference to a female as the one who helped her obtain drugs. These individuals served as a connection to drugs, but they also exercised power in that role to restrict access. For instance, one woman describes the barriers she faced from her peers in obtaining heroin for her first injection:

*“There's all kinds of drugs there, but it's like family status out there. We're all a bunch of homeless kids…they make it a total pain in your ass to get any new drugs there…I spent all day looking for it. ‘Cuz it's a small town. It's a closed community, and like hella kids there are strung out. We're all like really good friends but nobody would give me a fucking bag [of heroin], you know? And when I finally go the goddamn bag, nobody would give me a needle. So I had to run around and buy one from somebody.”*

This woman reported that she ultimately gave money to her boyfriend, who purchased heroin for her from a female friend.

 The frequency with which women paid for their drugs increased for the first injection of ketamine, such that about three in ten paid for it. Given that an average of two years elapsed between the first injection event of any drug and the first injection of ketamine, women may have had time to form more connections through which they could obtain drugs. However, the proportion of women who said that the injection was planned decreased in comparison to the first injection event (23% versus 53%), suggesting a concurrent increase in the spontaneity of their drug use. Several women gave accounts of receiving unsolicited offers of ketamine, or buying or being given ketamine when they were looking for some other drug:

*“It was free… it was there. We were like “Hey, spare any change?” cause he kind of looked like he had money, you know? He was like “I do not have any change, but I got a little K [ketamine].” And we were like “Can we have it?” And he was like “Sure.” And we were like, “OK.”…So we sat in the corner and fuckin jammed [injected] it while he watched.”*

### 3.4. Access to Syringes and Paraphernalia

At their first injection event, 30% of women accessed their syringes from a primary source, 60% obtained them from a secondary source, and 10% did not know where the syringe came from (Table 2). In contrast, for their first injection of ketamine, 50% obtained their syringe from a primary source, 47% from a secondary source, and only 3% did not know the origin of their syringe. Overall, reports of receptive syringe sharing (i.e., using a syringe after someone else) were low (3%) at the first injection event, but increased by the first injection of ketamine (13%). At both injection events most of the women said that the syringe they obtained from a secondary source (i.e., a source other than a needle exchange or pharmacy) was a brand new, sterile syringe that their friend or partner had procured from a needle exchange program: “My friend who I knew…he's a regular drug user. Like intravenous drug user, so he always has a few extras that are definitely clean. I trust him to make sure it's clean. I saw the package.” However, several other responses suggested that the origin of the syringes may be less certain for example, “they were pretty much regular users so I am assuming that they probably did use a needle exchange or something.”

### 3.5. Drug Preparation

A minority of women at both injection events controlled the preparation of their own drugs (20% at the first injection, 37% at first injection of ketamine; Table 2). Among those who did not prepare their own drugs for injection at the first injection event, almost half (46%) reported that a sexual partner prepared their drugs. In contrast, at the first injection of ketamine only 19% of women reported that a sexual partner prepared their injection. 

Notably, many women did not observe the preparation of the drug they injected. Women commonly said that they were not in the same room with the individual who was preparing their drugs and often were simply handed a filled syringe. For example, this woman recounts her first injection of ketamine, which was prepared by her husband: “He came back, he said, “I'm gonna go mix it up” and he went and mixed it up and he came and had my shot for me.”

While rates of syringe sharing were relatively low, almost two-thirds of women reported injecting with shared paraphernalia (i.e., cookers, cottons) at their first injection event (Table 2), and nearly three-quarters reported using shared paraphernalia (i.e., cookers, cottons, and ketamine vials) at the first ketamine injection. At the first injection event, a more experienced injector typically prepared enough for both people using a single cooker and a single cotton filter. Since the bulk of women reported acquiring new syringes from either primary or secondary sources, it is possible that the paraphernalia used in some of these shared injection events was new. Among the women who reported using shared cookers and cottons at their first injection event, almost all reported that someone else prepared the drug solution. In contrast, among those who used separate injection equipment, less than half reported that someone else prepared the drug.

At the first ketamine injection event, many women were faced with a novel drug form: liquid ketamine in a pharmaceutically manufactured, multi dose vial. Most of the women who shared injection equipment at the first ketamine injection reported sharing a multi dose vial. While several women reported that they used clean syringes to draw drug solution out of the vial, all participants did not. Similar to their first injection, almost all of the women who shared injection equipment had someone else prepare the drug. None of the women who used separate equipment reported that someone else prepared the drug for them.

### 3.6. Control over Injection

At the first injection event, a small proportion of women (17%) injected themselves (Table 2). Among those who did not inject themselves, most relied on friends (52%) or sexual partners (48%) to perform their first injection for them. Most women learned how to inject in the intervening time between their first injection and their first injection of ketamine; 80% of women performed their own first injection of ketamine. This is significant in light of the much lower proportion of individuals who prepared their own ketamine for injection (37%), suggesting that while most individuals gained control over the actual injection, many continued to rely on someone else for preparation. Among those who did not perform their own injection, two-thirds of women relied on a friend, while one-third of women had a sexual partner perform the injection. No women reported being injected by a stranger.

Most women said the primary reason that they performed their first ketamine injection themselves was that they were now familiar with how to administer an intravenous or intramuscular injection. “Once I learned how to hit, like, I learned how to use needles, I was pretty adamant about (injecting myself).” However, another woman said that she only injected herself that time because her boyfriend was already too high to do the injection for her, suggesting that her preference would have been to have him do it. As was the case with drug preparation, for some individuals the injection of ketamine for the first time required learning a new skill. Women described two modes of administration of the first shot of ketamine: intravenously (57%) and intramuscularly (43%). Women who self-administered their first injection of ketamine were more likely to have been injected intravenously (54%) versus intramuscularly (46%). Again, this association may be due to the fact that intravenous administration was a more familiar mode than intramuscular. 

## 4. Discussion

The social context of drug injection and associated risk for HIV and HCV among female IDUs is particularly relevant as some scholars have described the “feminization” of the HIV epidemic in recent years [29]. In this study, we asked a sample of young female IDUs to describe the social context of two pivotal events in their injection careers: their first injection of any drug and their first injection of a new drug (i.e., ketamine), which occurred an average of two years later. We found important differences in several aspects of the social environment that have implications for HIV/HCV risk in this group of high-risk women.

The group of IDUs with which women injected became larger and more diverse over the two years between injection events. This included an increase in different relationship types, including more strangers or casual acquaintances. As women matured in their drug using careers and became more confident, they may have also become more integrated into larger and more diverse drug using networks. This type of change in drug using social networks has implications for HIV/HCV risk, as introduction of new members of discordant or unknown serostatus may change the risk profile of the entire network [30, 31]. The prominent role of women's sexual partners in the events described here is consistent with the finding that young women are more likely than men to initiate injection with a sexual partner and to have that individual perform the injection [13, 32, 33]. It also illustrates the overlap in drug using and sexual networks (i.e., multiplexity) that has been shown to increase risk for HIV and HCV infection [3, 4]. 

Women's access to drugs was often facilitated by other people and women seldom paid for their own drugs. For many women, ketamine was encountered spontaneously and unexpectedly, usually offered for free by others in the using group. Dependence on others for access to drugs, which can result from women's marginalized positions in their drug-using social networks [8], could create scenarios in which women are unable to demand the use of separate, sterile injection equipment. Spontaneous drug injection may also create scenarios in which sterile injection equipment is unavailable. Both of these situations could elevate the likelihood that contaminated injection equipment is used, thereby increasing risk for HIV/HCV infection.

Women appeared to increase their ability to access syringes from a primary source over time. Increased access may be a product of women's maturation in their drug using career and increasing familiarity with community resources for syringes. Still, only half of women reported that they obtained their syringe from a primary source for their first ketamine injection. Women were not always certain that the syringes they obtained from secondary sources were sterile; therefore, obtaining syringes via secondary routes to engage in an unplanned injection event could have increased their risk for HIV/HCV infection. However, the risk associated with this strategy likely depends on the type of relationship and level of trust and communication about infectious disease between partners. In fact, procuring syringes from friends and/or sex partners may be a risk reduction strategy, particularly if the option is obtaining syringes from a stranger.

 Drug preparation is a critical point in the injection process during which contamination with bloodborne pathogens such as HIV or HCV can be introduced via contaminated cookers, cottons, or syringes, which are sometimes shared in order to prepare or divide drug solution [34]. Women were, to a large extent, dependent on others to prepare their drugs for them. In the current sample, women primarily relied on friends/acquaintances or sexual partners. Importantly, women who shared equipment at either injection event were more likely to have had someone else prepare the drug—those who used separate equipment tended to report that they prepared their own drug solution. The introduction of a new drug (ketamine) in unique forms (liquid and powder) could have increased the dependence on others. In several accounts of the first injection of ketamine, women said they did not witness the preparation of the drug, but they were willing to be injected with syringes prepared for them by others. Not seeing or having control over the preparation of one's drugs may dramatically increase the potential for cross-contamination and infection with HIV and/or HCV. 

Research suggests that many young people have others inject them for the first time [32], which was supported by our findings. After the first injection, most women learned to inject themselves, a step that vastly increases women's control over not only the sterility of the injection, but also over when and how much they inject. At the first ketamine injection, women who self-injected tended to inject intravenously, a mode with which they were already familiar, versus those who injected intramuscularly and required assistance. Nonetheless, at the first ketamine injection a substantial proportion (20%) still required or preferred that someone else inject them. Requiring help injecting or having someone else perform an injection has been statistically associated with HIV risk [12, 35]. Therefore, the remaining women who continued not to inject themselves are likely at increased risk of infection for as long as they continue to have someone else perform their injections. This situation also reinforces their dependence on their drug-using partners [8] and may elevate risk for drug overdose since they are not in control of the dosage of drugs.

 There were few reports of knowingly injecting with previously used syringes, and women commonly indicated that they knew that the use of previously used syringes was risky. The low frequency of reported syringe sharing could indicate a reporting bias induced by social desirability. However, it is also possible that this population of young injectors has grown up and begun injecting in an era of intense HIV and HCV prevention efforts, and therefore they actually have minimized syringe sharing, a phenomenon that has been reported elsewhere [13, 36]. A full 97% of women reported receiving at least one HIV test and 90% reported having had an HCV test in their lifetime, where they likely received some risk reduction counseling. Importantly, however, reports of sharing other injection paraphernalia (cookers, cotton, or ketamine vials) were frequent, a trend that has been identified as an independent risk for bloodborne pathogens [13]. Elevated rates of paraphernalia sharing among women have been observed elsewhere [33]. Our findings also suggest that it is possible that women had unknowingly used contaminated injection equipment. Additionally, few women had a thorough understanding of the dynamics of cross-contamination. Therefore, a lack of known sharing events may not reflect the use of new, sterile equipment for every injection.

### 4.1. Limitations

Due to the small sample size and nonrandom sampling techniques, it is difficult to know whether our findings are generalizable to the larger population of female IDUs. Reports of stigmatized behaviors such as syringe sharing may be subject to socially desirable reporting, and so the proportion of shared syringes may be under reported. The risk associated with shared paraphernalia in the current study may be overestimated; while we report that a large proportion of respondents shared some paraphernalia, in many accounts it was unclear whether the shared equipment was new at the time it was shared, or if it was previously used. Reports are also subject to recall bias, particularly given that, in some cases, several years had elapsed since the injection event. Due to the design of the study, participants reported a wide range of time elapsed between their first injection and their first injection of ketamine. Future research that systematically investigates these differences is needed. Finally, it is unknown whether the behavior reported at the first injection of ketamine is unique to the drug, or whether it can be generalized to injection initiation with other substances [20]. More investigation into the initiation of new substances among IDUs is warranted, and such investigations would benefit from longitudinal designs that are able to observe trajectories of drug use as they occur.

A strength of the current study is that it is one of the few investigations to focus on the unique risks faced by young female IDUs, see also [4, 33, 35], and that we were able to obtain reports of two injection events separated by an average of two years, which allowed us to examine the evolution of injection behaviors over time. The majority of existing investigations into the role of sex differences in injection-related risk have been conducted with significantly older populations [9, 11, 37–39], within which the dynamics of social relationships and habitual patterns drug use may be more established and differ from those of young women. The current analysis contributes to the growing body of literature that acknowledges the social nature of women's drug use and the unique challenges faced by female IDUs.

### 4.2. Conclusions

Our findings describe features of injection events in which women's lack of control may elevate their risk for infectious disease and suggest that these phenomena should be considered together as a behavioral risk factor, in addition to traditional indicators such as syringe and paraphernalia sharing. Current public health recommendations indicate that the most effective means of limiting HIV transmission among IDUs is the once-only use of sterile injection equipment [40]. Given the limited availability of injection equipment and clandestine nature of injection drug use, however, women take steps to reduce their risk for infectious disease in a less predictable environment that is contextualized by their social and intimate relationships. By understanding the dynamics of power and control over women's drug injection behavior within the social context, the current study has identified windows of opportunity to reduce the harm associated with drug injection. 

HIV and HCV prevention interventions tailored to address areas where women lack control over important aspects of their drug use, particularly the preparation of the drug and its injection, are an important next step in reducing HIV and HCV infections in this population. Our findings suggest that the spontaneous nature of injection events, and a lack of control over drug and syringe acquisition, preparation, and injection may elevate young women's risk for infectious disease. Additionally, interventions that focus on the mechanisms of cross-contamination are needed to assist young IDUs in making educated decisions about how and with whom they share drugs, particularly when they are confronted with new drugs or drugs in new forms.

## Figures and Tables

**Table 1:** Demographic characteristics of young female IDUs who inject ketamine (*N* = 30).

	*n* (%)
Median age	21
Race and ethnicity	
White/Caucasian	26 (86.7)
Black/African American	1 (3.3)
Hispanic/Latino	2 (6.7)
Asian or Pacific Islander	1 (3.3)
Native American	0 (—)
Multiracial background	0 (—)
Sexual identity	
Heterosexual	13 (60.0)
Gay/lesbian	0 (—)
Bisexual	12 (40.0)
Other/undecided	2 (6.7)
High school graduate or GED	19 (63.3)
Homeless	30 (100.0)
Employed full or part time	3 (10.0)
History of drug treatment	14 (46.7)
History of mental health care	20 (66.7)
Ever arrested	28 (93.3)
Ever in jail	25 (89.3)
Ever in prison	3 (10.7)
Tested for HIV	29 (96.7)
HIV positive (self-report)	0 (—)
Tested for HCV	27 (90.0)
HCV positive (self-report)	10 (33.3)

**Table 2:** Characteristics and social context of injection events among young female IDUs who inject ketamine (*N* = 30).

	First injection of any drug	First ketamine injection
	*n* (%)	*n* (%)
Type of drug		
Heroin	16 (53.3)	0 (—)
Ketamine	0 (—)	30 (100)
Methamphetamine	8 (26.7)	0 (—)
Cocaine	4 (13.3)	0 (—)
Other∗	2 (6.6)	0 (—)
Using group		
Median size (range)	2.0 (1–5)	2.0 (1–15)
Injected alone	2 (6.7)	2 (6.7)
Injection was planned	16 (53.3)	7 (23.3)
	(*n* = 29)	
Paid for drug	6 (20.7)	10 (33.3)
	(*n* = 29)	
Syringe source		
Primary	9 (30.0)	15 (50.0)
Secondary	18 (60.0)	14 (46.7)
Unknown	3 (10.0)	1 (3.3)
Injection equipment status		
Previously used syringe	1 (3.3)	4 (13.3)
Cleaned the syringe before use	0 (—)	3 (75.0)
Used equipment	19 (63.3)	22 (73.3)
Who prepared the drug?∗∗		
Self	6 (20.0)	11 (36.7)
Other	24 (80.0)	21 (70.0)
Friend/acquaint.	14 (58.3)	17 (81.0)
Stranger	0 (—)	0 (—)
Girlfriend/boyfriend	11 (45.8)	4 (19.0)
Family member	0 (—)	0 (—)
Who injected you with the drug?∗∗		
Self	5 (16.7)	24 (80.0)
Other	25 (83.3)	6 (20.0)
Friend/acquaint.	13 (52.0)	4 (66.7)
Stranger	0 (—)	0 (—)
Girlfriend/boyfriend	12 (48.0)	2 (33.3)
Family member	0 (—)	0 (—)

^*^Includes alcohol, speedball, Ritalin, morphine, and Dilaudid.

∗∗Categories are not mutually exclusive.
